# Real-time cardiovascular magnetic resonance subxiphoid pericardial access and pericardiocentesis using off-the-shelf devices in swine

**DOI:** 10.1186/1532-429X-15-61

**Published:** 2013-07-20

**Authors:** Majdi Halabi, Anthony Z Faranesh, William H Schenke, Victor J Wright, Michael S Hansen, Christina E Saikus, Ozgur Kocaturk, Robert J Lederman, Kanishka Ratnayaka

**Affiliations:** 1 Cardiovascular and Pulmonary Branch, Division of Intramural Research, National Heart Lung and Blood Institute, Bethesda, MD 20892-1538, USA; 2 Department of Cardiology, Children’s National Medical Center, Washington, DC, USA

**Keywords:** Pericardiocentesis, Catheterization, Image guided intervention, Interventional magnetic resonance imaging, Cardiovascular magnetic resonance, Pericardial disease

## Abstract

**Background:**

Needle access or drainage of pericardial effusion, especially when small, entails risk of bystander tissue injury or operator uncertainty about proposed trajectories. Cardiovascular magnetic resonance (CMR) might allow enhanced imaging guidance.

**Methods and results:**

We used real-time CMR to guide subxiphoid pericardial access in naïve swine using commercial 18G titanium puncture needles, which were exchanged for pericardial catheters. To test the value of CMR needle pericardiocentesis, we also created intentional pericardial effusions of a range of volumes, via a separate transvenous-transatrial catheter. We performed these procedures in 12 animals.

Pericardiocentesis was performed in 2:47 ± 1:43 minutes; pericardial access was performed in 1:40 ± 4:34 minutes. The procedure was successful in all animals. Moderate and large effusions required only one needle pass. There were no complications, including pleural, hepatic or myocardial transit.

**Conclusions:**

CMR guided pericardiocentesis is attractive because the large field of view and soft tissue imaging depict global anatomic context in arbitrary planes, and allow the operator to plan trajectories that limit inadvertent bystander tissue injury. More important, CMR provides continuous visualization of the needle and target throughout the procedure. Using even passive needle devices, CMR enabled rapid pericardial needle access and drainage. We believe this experience supports clinical testing of real-time CMR guided needle access or drainage of the pericardial space. We suspect this would be especially helpful in “difficult” pericardial access, for example, in distorted thoracic anatomy or loculated effusion.

## Background

Pericardiocentesis is a common catheter technique to sample or drain fluid from the pericardial space [1]. Pericardial access may be desirable even without effusion in certain procedures such as epicardial ablation of rhythm disorders [2], or atrial appendage exclusion [3]. Pericardiocentesis is performed using a long subxiphoid or transthoracic needle. Pericardiocentesis may be guided (A) using surface landmarks (“blindly”), (B) using electro-grams to detect epicardial needle contact, (C) using echocardiography, (D) using X-ray with- or without- contrast injections, or (E) using a combination. Each has advantages and limitations.

Typically, adjunctive imaging decreases procedural risk for difficult-to-access pericardial fluid collections (smaller volume, posterior location, loculated fluid, compartmentalized fluid collections, thick or calcified pericardium, large body habitus, etc.). In practice, most clinical techniques focus more on trajectory planning than on guiding live needle advancement to achieve pericardial access without bystander injury. Planar echocardiography imaging rarely allows the operator to view needle, neighboring vital structures, and target effusion simultaneously. Projection fluoroscopic imaging provides a large field of view, but uncertainty about three-dimensional position of soft tissue. Enhanced imaging guidance would be desirable.

We [4] and others [5-9] use real-time cardiovascular magnetic resonance (CMR) to guide clinical heart catheterization by tissue and chamber visualization but without X-ray radiation. We hypothesize CMR can also guide pericardiocentesis. Real-time CMR depicts the entire thoracic context for needle access, allowing the operator to avoid critical structures including the liver, lung, pleural space, and heart muscle. We evaluate the safety and feasibility of real-time CMR guided needle pericardiocentesis in animals with and without pericardial effusions, using commercially available CMR-compatible (“passive”) access needles.

## Methods

### Animal model

Animal procedures were approved by the institutional animal care and use committee, and followed contemporary National Institutes of Health guidelines. Twelve naïve Yorkshire swine (Animal Biotech Industries, Danboro, PA) were anesthetized with atropine, butorphanol, ketamine, and xylazine, and maintained on isoflurane with mechanical ventilation. Percutaneous arterial and venous access was obtained.

Four groups of three animals were tested, having either no effusion, small (50 ml), moderate (100 ml), or large (150 ml) effusions. Pericardial effusions of known volumes were created by infusing normal saline using separate transfemoral catheters that crossed the right atrial appendage into the pericardial space [10]. Catheters were placed under X-ray prior to CMR. After effusions were created, hemodynamics were recorded, CMR was performed, and drainage was attempted. The time interval between creating and draining effusions was approximately five minutes. Pericardial catheters were not placed in the “no effusion animals” to avoid distorting normal pericardial volume.

### Magnetic resonance imaging

Experiments were conducted in a combined interventional CMR and X-ray fluoroscopy suite (1.5T *Espree. Axiom Artis*; *Siemens*, Erlangen, Germany) detailed elsewhere [4]. Hemodynamics were recorded continuously. CMR headsets afford acoustic sound suppression and “open-microphone” communications among staff and on a second channel in case of an awake patient (*IMROC*, Opto-acoustics, Moshav Mazor, Israel). Interventional procedures were guided by real-time balanced steady-state free precession (bSSFP) and the following parameters: repetition time (TR) / echo time (TE), 2.88/1.44 ms; flip angle, 40°; bandwidth, 1000 Hz/pixel; field of view (FOV), 350 mm; matrix 144 × 192 pixels; and slice thickness, 6 mm, GRAPPA rate 1–2, with a temporal resolution up to 150 ms (7 frames/s). We used a prototype commercial real-time CMR user interface (*Interactive Front End*, Siemens Corporate Research, Princeton) for trajectory planning in multiple planes and for imaging guidance of the interventional procedure.

Pericardial effusion size was measured using bSSFP CMR with the following typical ECG triggered, breath-held parameters: TR/TE, 3/1.3 ms and partial-echo readout; flip angle, 65°; bandwidth, 1028 Hz/pixel; fat-saturation prepulses, FOV, 344 mm; matrix, 256 × 256 pixels; slice thickness, 10 mm; 10 slices (axial, coronal, sagittal).

### Real-time CMR guided pericardiocentesis

**Figure 1:**
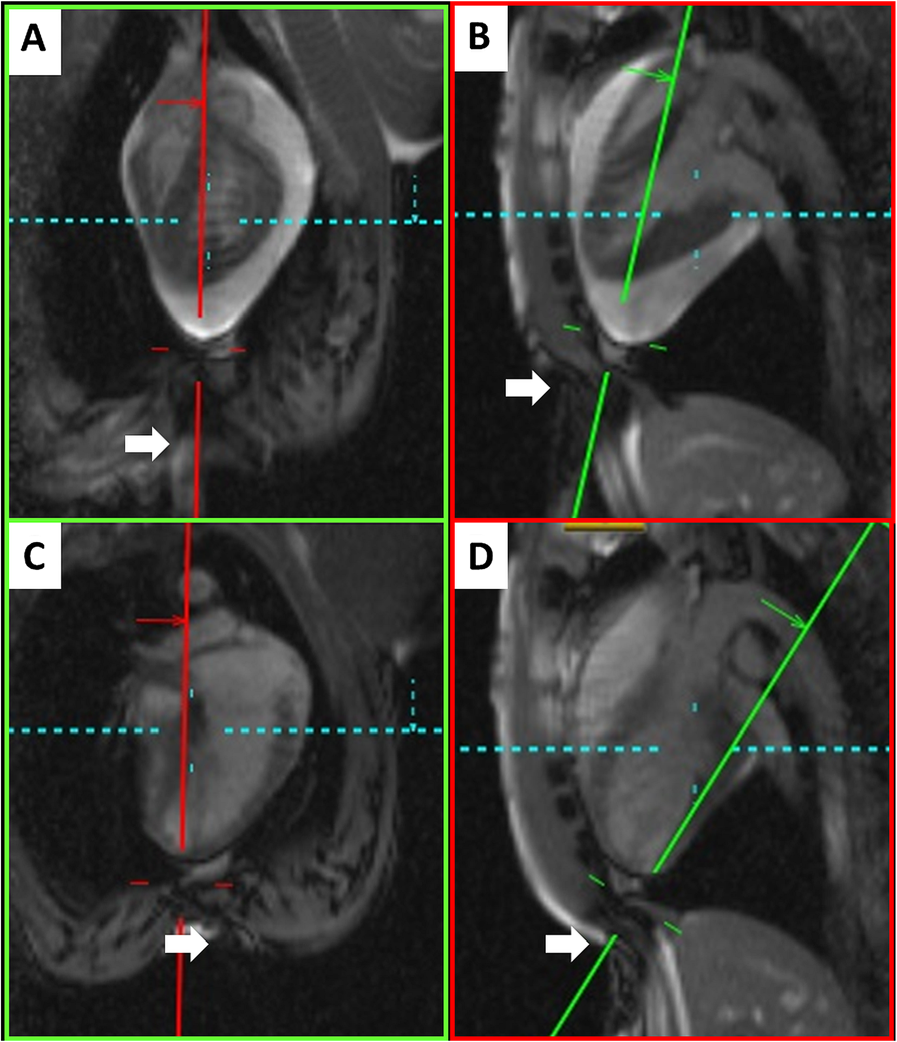
**Trajectory planning: Real time CMR pericardiocentesis.** Real-time CMR system user interface (Interactive Front End, Siemens Corporate Research, Princeton) is used for trajectory planning. The prescribed slice planes intersect along the projected needle path. The operator finger (white arrow) depresses the subxiphoid skin surface to mark the proposed needle entry site. Panel **A** (coronal) and **B** (sagittal) is an animal with a large (150 ml) effusion. Panel **C** (coronal) and **D** (sagittal) is an animal with no effusion. Posterior trajectory is chosen. The blue dotted line indicates an axial imaging plane prescription that is not shown.

Subxiphoid needle entry and trajectory were planned on real-time images, typically oblique coronal and sagittal views (Figure 1). We used CMR compatible needles marketed in the US (*CMR Puncture Needle*, 18G × 15 cm, Philips Invivo, Schwerin, Germany).

The needle was advanced using real-time CMR guidance to maintain a planned trajectory. In animals having an effusion, pericardial entry was confirmed using CMR. In these animals, pericardial entry was corroborated by tactile feedback, ectopic heart beats, and fluid aspiration.

In animals having no effusion (“dry tap”), the pericardium was entered and corroborative test injection of saline (10–20 ml) through the needle was performed using real-time CMR. Pericardial access was confirmed by instilling saline after a pericardial “pigtail” catheter was introduced over a nitinol guidewire. Animals were observed for 60 minutes after the procedure to detect complications.

### Data analysis

We recorded success or failure, number of needle attempts, and complications. We recorded the time required to perform certain procedure elements, including CMR time required to plan the trajectory, and time required to access the pericardial space after the needle crossed the skin. Hemodynamics were reported before and after intervention. Effusions were measured by maximal dimension in three planes (axial, coronal, and sagittal).

Results are expressed as mean ± standard deviation, and where appropriate, 95% confidence interval or range. Time to perform procedure steps are expressed as median and range. Continuous parameters were tested using analysis of variance and Tukey’s correction for multiple comparisons. The numbers of discrete needle attempts for different sized effusions were compared using a Kruskal-Wallis test with Dunn's correction for multiple comparisons. Data were analyzed using Microsoft Excel or Graphpad Prism (v5.04). A p < 0.05 was considered significant.

## Results

### Twelve animals (41.1 ± 3.5 kg) were studied

No pericardial separation was evident in the animals without baseline effusion. Small (50 mL) effusions had maximal two-dimensional pericardial separation less than 10 mm. Moderate (100 mL) effusions measured between 10-20 mm, and large (150 mL) effusions were approximately larger than 20 mm (Table 1).

**Table 1:** Pericardial effusion size

**Effusion size (volume infused)**	**Drained volume *ml***	**Pericardial pressure *mmHg***	**Maximum dimension *mm *coronal**	**Maximum dimension *mm *sagittal**	**Maximum dimension *mm *axial**
**Small (50 ml)**	48.4 ± 3.0	3.4 ± 1.2	9.5 ± 2.4	9.4 ± 2.1	8.6 ± 0.7
**Moderate (100 ml)**	98.3 ± 3.7	6.1 ± 1.1	16.7 ± 3.5	16.6 ± 3.3	12.4 ± 1.6
**Large (150 ml)**	146.3 ± 4.8	4.8 ± 0.7	20.8 ± 3.8	22.1 ± 3.6	15.9 ± 2.2

Volumes are expressed in milliliters (ml). The units for pressure are millimeter of Mercury (mmHg). Maximum dimensions are represented in millimeters. n = 12 per group.

**Figure 2:**
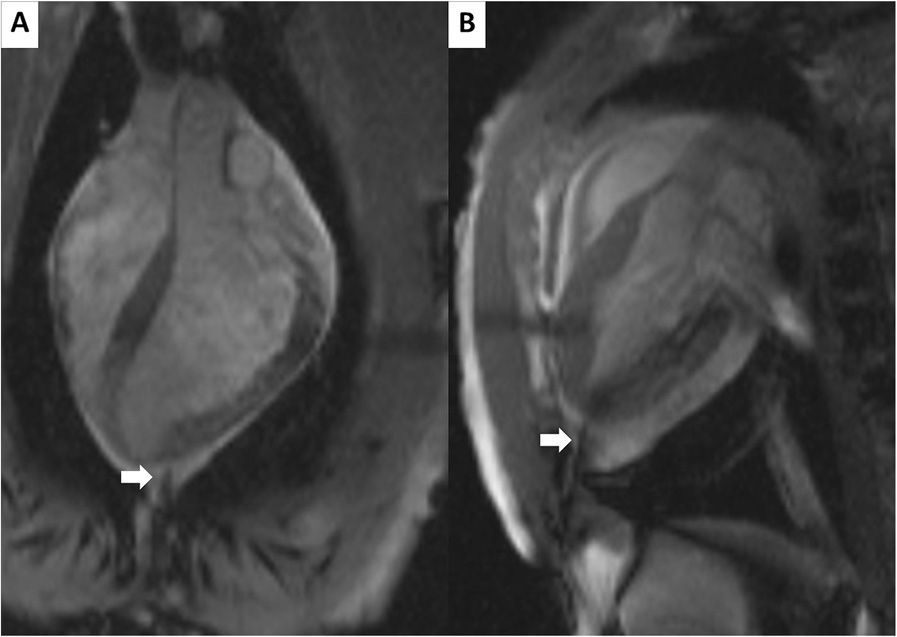
**Real time CMR guided pericardiocentesis using a “passive” needle.** Real time CMR guides pericardiocentesis performed with commercially available, off-the-shelf needle (white arrow). Panels **A** and **B** show typical long-axis imaging planes selected by the operators. This animal has a small (50 ml) effusion. Note the “saturation band” of reduced T1 recovery (and reduced signal) where the two imaging planes intersect.

For procedure guidance, we used non-standard cardiac oblique planes such as four-chamber and three-chamber long axis views, tailored for the maximum evident effusion. Because CMR depicts a broad anatomic context, we found it equally easy to access anterior and posterior pericardial spaces (Figures 1 &2).

Real-time CMR pericardial needle entry was successful in all. CMR guided pericardiocentesis was successful in all animals evident by fluid evacuation or purposeful accumulation (no effusion group).

*Trajectory planning time* took approximately one minute or less. Moderate and large effusions all required only one needle pass (p < 0.05 compared with no effusion). Small effusions required 2.7 ± 1.2 passes (p = NS compared with no effusion). Access without effusions required 3.3 ± 1.2 passes. Larger effusions required numerically but not statistically shorter access and procedure times (Table 2).

**Table 2:** Procedural details

**Effusion size**	**# of needle attempts**	**Trajectory planning time *min***	**Access time *min***	**Total procedure time *min***
**None**	4 (2–4)	1:03 (0:37–2:50)	8:21 (1:40–13:45)	9:24 (4:30–14:22)
**Small (50 ml)**	2 (2–4)	0:36 (0:27–0:46)	6:03 (2:29–9:38)	6:40 (2:56–10:24)
**Moderate (100 ml)**	*1	0:49 (0:44–1:35)	1:09 (0:24–1:11)	2:00 (1:08–2:44)
**Large (150 ml)**	*1	1:04 (0:44–1:50)	0:57 (0:23–1:54)	2:38 (1:27–2:47)

Values are median and range. * = p < 0.05 compared with no effusion. n = 3 per group.

**Figure 3:**
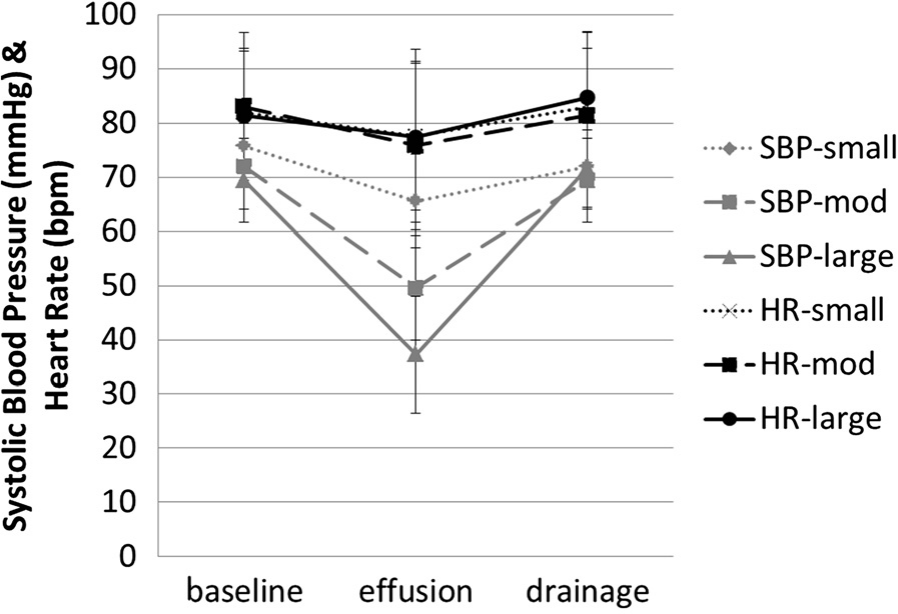
**Hemodynamics.** Systemic peak systolic blood pressure (SBP; mmHg) and heart rate (HR; bpm = beats per minute) are charted for small, moderate (mod), and large pericardial effusions at baseline, after the effusion is created, and after drainage. All effusions caused hemodynamic changes that returned to baseline after drainage.

Acute effusions decreased blood pressure. Heart rates did not increase. Drainage restored hemodynamics to near baseline (Figure 3). Cardiac tamponade physiology (systemic systolic blood pressure decrease of 15%) [11] was elicited with even small (50 mL) effusions (13.1% ± 10.6%, p < 0.05; 95% confidence interval of difference = 2.2 mm Hg to 18.1 mm Hg). Bigger (100 or 150 mL) effusions produced progressively more dramatic compromise (moderate, 30.5% ± 15.1%, p < 0.05; 95% confidence interval of difference = 13.9 to 30.9; and large, 46.2% ± 14.1%, p < 0.05; 95% confidence interval of difference = 23.5 to 40.9 respectively).

None of the following known complications of pericardiocentesis were observed: sustained dysrhythmia, coronary or thoracic wall vessel puncture, ventricle laceration, hemothorax, pneumothorax, pneumopericardium, hepatic injury, death [12].

## Discussion

We found that real-time CMR guided pericardial access and pericardiocentesis was successful in every attempt. This included animals with small and no pericardial effusions. Procedures were quick with no complications. As expected, larger effusions were easier and faster to access. This was accomplished using off-the-shelf commercial CMR needles marketed in the US.

Pericardiocentesis can be performed without imaging guidance, such as when death is otherwise imminent. Imaging guidance may reduce complications (procedure failure; coronary, myocardial or hepatic laceration; sustained arrhythmia, *etc.*). X-ray fluoroscopy projection has limited ability to distinguish pericardial from myocardial boundaries, and is poor at depicting three-dimensional position. Ultrasound may interfere with operator access, may be limited by acoustic windows, and has a limited field of view that may not depict the global anatomic context. By contrast, CMR combines superior soft tissue visualization, a larger field of view, multi-planar capability, and continuous visualization of the needle and target throughout the procedure.

In this pre-clinical experience, we found real-time CMR guidance useful. CMR visualization of device, trajectory, and target enhanced operator subjective confidence in procedure steps. Objectively, pericardial access was achieved in all experiments without complications, irrespective of pericardial fluid volume, even in this early-stage experiment. The temporal resolution was adequate to guide the procedure in these animals with clinically-relevant tachycardia. Our success in animals having no effusion suggests potential value in other procedures requiring pericardial or epicardial access. The large field of view afforded by CMR may facilitate alternative, unconventional (mid-clavicular, mid-axillary) approaches to drain non-circumferential, difficult-to-access pericardial fluid [13], or very small pericardial effusions [14]. We only evaluated subxiphoid access because of the subxiphoid orientation of the porcine cardiac apex; in other work we accessed the porcine heart and pericardial space via intercostal approaches [15,16].

**Figure 4:**
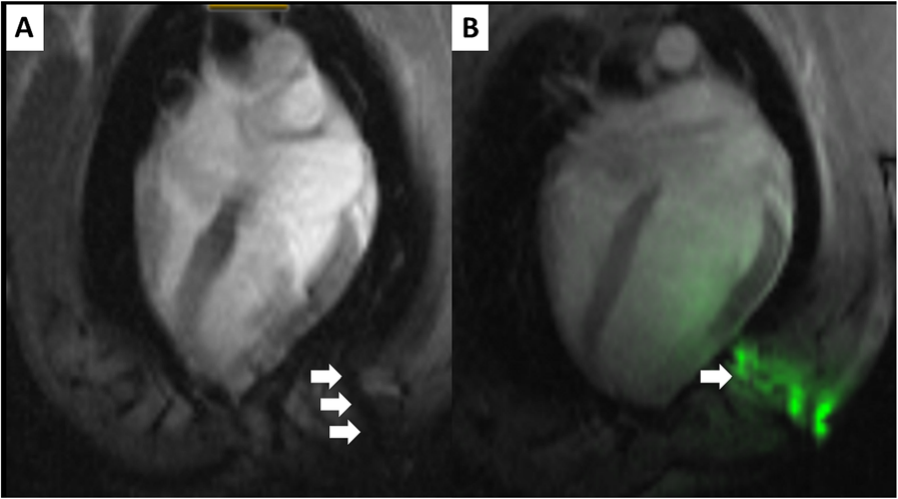
**Comparison of “passive” versus “active” visualization of needles during CMR guided pericardial access.** The “passive” titanium needle (panel **A**, black artifact indicated with white arrows) is compared with an “active” antenna needle (panel **B**, green colorized signal indicated with white arrow). The active needle is more conspicuous. This demonstration uses a lateral access approach and no pericardial effusion.

In this experiment, we showed good success using “passive” commercial CMR needles that are visualized based on intrinsic materials properties. In other work, we have shown superior visibility using an investigational “active” antenna-needle (Figure 4) [17]. Both may enable more sophisticated non-surgical treatments for structural heart disease, including direct transthoracic implantation of large appliances into the heart [16].

Several limitations are noteworthy. Few clinical CMR systems currently are configured for interventional procedures including in-room display, interactive control, acoustic sound compression, open-microphone communications systems, and high-fidelity hemodynamic monitoring, although these represent a small incremental increase in cost. Even large-bore CMR systems constrain access to the chest or subxiphoid space, although we have found this readily surmountable even for sterile procedures in our preclinical experience and in human volunteers [data not shown]. Although we used clinical CMR needles, there are currently no commercially available safe and conspicuous CMR guidewires to be used in tandem with our access needle. Iron-doped polymer passive devices have shown mixed performance in early development [7]. We chose to use interleaved multiplanar CMR which creates a “saturation” artifact along their line of intersection that usually coincides with the desired needle trajectory (see Figure 2); this further limits the visibility of passive needles except when single-plane imaging is selected interactively. Passive needles are difficult to visualize outside the body before the operator commits to a needle trajectory. We believe active devices may enhance visibility and safety, and therefore we are developing clinical-grade active needles [17] and guidewires [18]. Finally, interventional CMR remains investigational and is not yet widely deployed.

## Conclusion

In conclusion, we found real-time CMR allowed successful and rapid pericardial entry and pericardiocentesis in swine irrespective of pericardial volume, using off-the-shelf needle devices. Clinical testing is underway.

## Abbreviations

FOV: Field of view; CMR: Cardiovascular magnetic resonance; SSFP: Steady state free precession; TE: Echo time; TR: Repetition time.

## Competing interests

NIH and Siemens Medical Systems have a collaborative research and development agreement for interventional cardiovascular MR.

## Authors’ contributions

MH conceived the study, performed and analyzed the experiments, and edited the manuscript. AZF, WHS, VJW, MSH assisted in experimental design and conduct. CES and OK designed and tested custom and standard needles. RJL assisted in experimental design and analysis, and edited the manuscript. KR conceived the study, assisted with the experiments, participated in the analysis, and drafted the manuscript. All authors read and approved the final manuscript.
